# Comparison of antibacterial effectiveness of three rotary file system with different geometry in infected root canals before and after instrumentation–a double-blinded randomized controlled clinical trial

**DOI:** 10.1038/s41405-020-0035-7

**Published:** 2020-06-08

**Authors:** Riluwan Siddique, Malli Sureshbabu Nivedhitha, Manish Ranjan, Benoy Jacob, Pradeep Solete

**Affiliations:** Saveetha Dental College, Poonamalle High Road, Chennai, Tamil Nadu 600056 India

**Keywords:** Endodontic files, Root canal treatment

## Abstract

**Introduction:**

To compare the antibacterial effectiveness of three rotary file systems i.e., ProTaper Next, ProTaper Gold and XP-endo Shaper in root canals of teeth with asymptomatic apical periodontitis by using the real-time polymerase chain reaction.

**Materials and methods:**

Root canals from single or multi-rooted teeth (straight canals) with necrotic pulps and asymptomatic apical periodontitis were instrumented using either ProTaper Next (*n* = 20), ProTaper Gold (*n* = 20) and XP-endo Shaper (*n* = 20) under irrigation with 3% sodium hypochlorite. Samples obtained before and after instrumentation were subjected to DNA extraction, amplification and quantitation of total amount of bacteria by using the real-time polymerase chain reaction.

**Results:**

Samples were taken before preparation (S1) were positive for presence of bacteria, with mean numbers of 9.94 × 10^7^, 20.4 × 10^7^ and 9.20 × 10^7^ bacterial cells for the ProTaper Next, ProTaper Gold and XP-endo Shaper groups, respectively. After preparation (S2) with ProTaper Next, ProTaper Gold, and XP-endo Shaper, root canals still had bacteria with mean counts of 11.8 × 10^5^, 87.2 × 10^5^ and 4.52 × 10^5^ bacterial cells, respectively. Both XP-endo Shaper (99.50%) and ProTaper Next (98.81%) were effective in reducing total bacterial count, and there was no statistically significant difference between them (*P* > 0.05). XP-endo Shaper succeeded in reducing total bacterial count than ProTaper Gold (95.72%) and there exists statistically significant difference between them (*P* < 0.05).

**Conclusions:**

XP-endo Shaper was highly effective in reducing total bacterial count from root canals of teeth with asymptomatic apical periodontitis than ProTaper Gold. ProTaper Next also showed improved microbial reduction percentage as compared with ProTaper Gold.

## Introduction

The main goal of endodontic treatment is to reduce the number of bacteria and their byproducts which perpetuates apical periodontitis.^1^Effective chemo-mechanical debridement techniques on par with stringent disinfection protocols aid in possibly diminishing the bacterial load residing in the complexities of the root canal systems.^2^

Several mechanical devices and techniques were introduced to make canal preparation easier and to further improve the effectiveness of instrumentation.^3–5^ The devices for root canal instrumentation may be classified as either manual or machine assisted. Machine-driven endodontic instruments help to prepare the root canals swiftly with much ease as opposed to manual instrumentation.^6^ Diverse machine-assisted techniques include automated, sonic and ultrasonic, laser systems and non-instrumental root canal preparation techniques.

The ProTaper Next (2013) was made utilizing M-Wire technology which incorporates five files (X1–X5). The files are inbred with tapers of 0.04 mm, 0.06 mm, 0.07 mm, 0.06 mm, and 0.06 mm, respectively. An off-centred rectangular cross-section design imparts improved file strength with unique asymmetric rotary motion that further enhances ProTaper canal shaping efficiency and cyclic fatigue resistance.^7–9^ This property allows the file to maintain a two-point contact with each canal at a time. In accordance with a study by Tewari et al., ProTaper Next was found to be most effective in microbial reduction than K3XF, Hyflex CM and hand instruments.^10^ ProTaper Next showed maximal microbial load reduction than self-adjusting files and manual instrumentation.^11^ To sum it up, the PTN system reinforces file strength, capable of reducing lateral and apical compaction of debris with more efficient cleansing of the root canal system.

The ProTaper Gold (2014) was fabricated using gold heat treatment technology. It has convex triangular cross-section and progressive taper which enhances cutting action while decreasing rotational friction between the blade of the file and dentin.^12^ PTG instruments produced less transportation; maintained more dentin than ProTaper Universal (PTU).^13^ During the manufacturing phase, the files are subjected to a unique heat treatment process ultimately exhibiting different phase transformation behaviour, higher flexibility, and higher cyclic fatigue resistance. The physical properties mentioned above are most suited for preparing root canals with challenging abrupt curvatures.^14^

XP-endo Shaper (XP) was introduced in 2015 which claimed progressions of new rotary NiTi system created with MaxWire technology. It is a snake-shaped instrument which has a triangular booster tip cross-sectional design with six sharp cutting edges at the tip and an ISO 15 (0.01 taper) initial diameter which increases to ISO diameter 30 (0.04 taper). It works by eccentric rotary motion by taking on a semicircular shape. It is in martensitic phase before being introduced into the root canals, changes its shape as a result of molecular memory of austenitic phase. It has been proved that the XP-endo Shaper follows the root canal anatomy of oval shaped canals by preparing and touching more walls than the Vortex Blue system, iRaCe and Edgefile system.^15,16^ Various in vitro studies claimed that XP-endo Shaper induces minimal stress on dentinal walls, thereby preventing formation of new dentinal cracks and also effective in reducing microbial count levels in oval shaped canals.^17–23^

However, there is lack of clinical studies to prove the effectiveness of three-dimensional rotary systems over traditional files. A recent systematic review of clinical trials (Siddique and Nivedhitha, 2019) demonstrated that there was no significant difference between rotary as well as reciproc system on microbial reduction.^24^ This clearly indicates the need for determining the effect of three-dimensional rotary system in clinical trials.

Therefore, the aim of this double-blinded randomized controlled clinical trial was to compare the microbial load reduction of ProTaper Next, ProTaper Gold and XP-endo Shaper in infected root canals of teeth with asymptomatic apical periodontitis. The null hypothesis was that there was no statistically significant difference in microbial load reduction between three rotary systems.

## Materials & methods

This clinical trial compared the effectiveness of three rotary file systems namely ProTaper Next (PTN, Dentsply Maillefer, Ballaigues, Switzerland), ProTaper Gold (PTG; Dentsply Maillefer, Ballaigues, Switzerland) and XP-endo Shaper (FKG Dentaire, La Chaux-de-Fonds, Switzerland) which followed the guidelines of the revised Consolidated Standards of Reporting Trials statement.^25^

### Study design and ethical approval

It was a prospective double-blinded randomized controlled clinical trial. The study protocol was approved by the Institutional Human Ethical Committee of University [IHEC Ref No: SDC/ENDO-18/0119] and informed consent was obtained from all patients. The clinical trial was registered in Clinical Trials Registry-India (CTRI) with reference number CTRI/2019/04/018540. Sample collection was performed by single well-trained operator throughout the completion of study.

### Sample size determination

Sample size calculation was performed based on the results of pilot study with 5 samples in each group which is done before commencement of this clinical trial. The sample size was found to be 5 in each group (allocation ratio 1:1:1) using G*Power 3.1.9.4 with alpha error left at 5% and statistical power of 95%. All 5 samples per group used in pilot study were included in this clinical trial.

### Setting and location

Samples were taken from 60 patients (24 males and 36 females; mean age of 36 years, ranging from 18 to 75 years) who were recruited from the pool of patients in the Department of Conservative Dentistry and Endodontics, Saveetha dental college, Chennai.

### Case selection

Sixty patients were assigned to this double-blinded randomized controlled clinical trial with indication for root canal treatment. Inclusion criteria were as follows: asymptomatic apical periodontitis, single & multi-rooted teeth with straight canals, intact pulp chamber walls, necrotic pulps confirmed by pulp tests and, clinical and radiographic evidence of asymptomatic apical periodontitis. In multi-rooted teeth, palatal and distal canals of maxillary and mandibular molars were included. Teeth with gross carious lesion, crown or root fracture, retreatment, periodontal pocket deeper than 4 mm were excluded.

Out of 60 root canal samples, 42 single rooted and 18 multi rooted tooth samples were included in this clinical trial.

### Randomization, allocation concealment and blinding

Randomization was done well in advance by a third person who was not related to the study. Randomization was done using block randomization procedures using random number table with block sizes being unknown to the investigators until the completion of the study. SNOSE (sequentially numbered, opaque, sealed envelopes) method was implemented for allocation concealment which conceals the sequence until interventions were assigned. A piece of paper containing randomized group number was sealed in the dark-coloured envelope containing respective serial number over it which was prepared by a third person. The treatment protocol is also mentioned clearly and sealed in the envelope. Study numbers were sequentially assigned to patients. The envelope was opened once the intervention was assigned. Respective treatment was carried out based on the group assigned in the paper as described later. The study was double blinded as the patient and evaluator were blinded to the type of intervention being used.

### Sample collection and treatment procedures

Samples from root canals of teeth with asymptomatic apical periodontitis were collected under strict aseptic conditions. Patients were given oral prophylaxis (0.12% chlorhexidine followed by removal of supragingival biofilm by scaling) before initiating treatment. Rubber dam isolation was done followed by operative field including tooth, clamp, and surrounding dam were cleaned by using 3% hydrogen peroxide and then disinfected with 3 % sodium hypochlorite (NaOCl). Access cavity preparation was completed with the help of sterile burs under sterile saline irrigation. Disinfection was done using 2-step disinfection protocol with the sequential use of 3% hydrogen peroxide and 3% NaOCl. Next step was to inactivate residue of sodium hypochlorite by using 5% sodium thiosulphate and sterility control samples were taken by scrubbing against the cavosurface angle of the access cavity with the help of sterile paper points (Dentsply Maillefer). For the consideration of tooth in study, the sterility control tests must be negative for microorganisms in an end point polymerase chain reaction (PCR) assay by using universal bacterial primers.

Samples were taken from the root canal immediately before instrumentation (S1 sample) and after instrumentation (S2 sample). Sodium thiosulfate solution was placed in the pulp chamber without overflowing, and a small instrument was used to carry the solution into the canal. The root canal walls were gently filed with a small instrument so as to suspend the canal contents in sodium thiosulfate solution. Sterile paper points were consecutively placed in the canal to a level of ~1 mm short of the radiographic root apex, based on diagnostic radiographs, and each paper was kept in canal for about 1 min to soak up the fluid in the canal. S1 samples were collected using paper points which were suspended in 1.5 ml sterile DNase/RNase free tube containing 50 µl of 10% SDS (Sodium Dodecyl Sulfate) (Cat#194831, MP Bio, Canada) and 10% Triton X100 (Cat#64518, SRL Chemicals, India). From 50 µl of lysates, 1 µl was used to estimate the DNA concentration. All samples were diluted subsequently to 0.2 ng per µl, which were then used for experiments.

After irrigation with 1 mL of 3% NaOCl, the working length was determined by using an electronic apex locator (RZX; J Morita, Tokyo, Japan) and confirmation with radiographs.

Teeth were randomly allocated into three groups (*n* = 60) according to the rotary system used for root canal preparation. The shaping and cleaning of root canal system were completed in single visit followed by obturation in next visit.

### Protaper next group

Thirteen single rooted and seven multi rooted tooth samples were included in this group. Root canals were prepared by using the ProTaper Next (PTN, Dentsply Maillefer, Ballaigues, Switzerland) operated in continuous rotation motion at 300 rpm and a torque of 5.2 Ncm by an electric motor (X-Smart Plus motor, Dentsply Maillefer). The final preparation was done using master apical file size of X3. Patency of the apical foramen was confirmed with a size of 25 K-type hand file throughout the treatment procedures. Root canal preparation was done with 3% sodium hypochlorite floating in canal.

### Protaper gold group

Fourteen single rooted and six multi rooted tooth samples were included in this group. Root canals were prepared by using the ProTaper Gold (PTG; Dentsply Maillefer, Ballaigues, Switzerland) operated in continuous rotation motion at 300 rpm and a torque of 5.2 Ncm by an electric motor (X-Smart Plus motor, Dentsply Maillefer). Hand instrumentation was done till size of 25 K -type hand file. The root canal preparation was done in presence of NaOCl “Floating” with files ranging from SX to S2. The final root canal preparation was done using finishing file F3(ISO diameter 30) with a brushing action according to canal anatomy.

### XP-endo shaper group

Fifteen single rooted and five multi rooted tooth samples were included in this group. Root canals were prepared by using XP-endo Shaper (FKG Dentaire, La Chaux-de-Fonds, Switzerland) operated in continuous rotation motion at 800 rpm and a torque of 1 Ncm by an electric motor (X-Smart Plus motor, Dentsply Maillefer). Glide path of at least 25/.02 was used before using XP Endoshaper (30/.04) in root canals. It is used with gentle strokes until working length is reached and care was taken to avoid pecking motion. If working length is not reached with gentle five strokes, recapitulation was done with the help of irrigant and repeated. XP-endo Finisher was used as the final step for 1 min for active irrigation (~60 strokes).

During instrumentation, EDTA gel (Anabond Endoprep-Rc) was used as a lubricating agent for easy instrumenting of canals with endodontic files.

Finally, in all groups, the smear layer removal was done by using 5 mL of 17% EDTA (META BIOMED CO.LTD) and 5 mL of 3% NaOCl after biomechanical preparation of root canal. In each group, total volume of 15 mL NaOCl was used. In each group, total volume of 15 mL NaOCl was used. Sterile paper points were used to dry canals and then flushed with 5% sodium thiosulphate for 1 min for inactivation of NaOCl. A postoperative sample (S2) were collected carefully from the root canals.

Calcium hydroxide (RC Cal; Prime Dental Products Pvt. Ltd., Thane, India) was used as an intracanal medicament for 1week followed by obturation the following week.

### DNA extraction^26^

The samples were ^26^heated at 95 °C for 30 min, at the time of DNA extraction and centrifuged at 10,000 rpm for 3 min at room temperature to pellet the debris. The final supernatant containing the DNA were transferred to a fresh 0.5 ml sterile DNase/RNase free tube. The Qubit fluorometer (Life Technologies, USA) was used to quantify the total amount of DNA present in each of the sample.

### Amplification and quantitation of total amount of bacteria

In order to quantify the total amount of bacteria in the samples, an equal concentration (0.1 ng) of total genomic DNA was subjected to real-time PCR amplification with a pair of hypervariable regions 16 S V3 specific primers:^27^

CCTACGGGAGGCAGCAG

ATTACCGCGGCTGCTGG

10 µM of each of the above primers were added to TB green RT-Master Mix (Cat# RR820L, Takara Bio, Japan) in 20 µl reaction, and samples were analysed in Rotor Gene Q real time PCR equipment (Qiagen, Germany). The following universal amplification condition was used: after an initial denaturation at 95 °C for 10 min, samples were amplified for 40 cycles at 94 °C for 25 s, 51 °C for 25 s, and 72 °C for 25 s.

### Establishment of standard

In order to quantitatively determine the copy numbers of each bacteria (relative to each other and among the samples), a standard curve was established with serial dilutions of PCR product amplified from V5 to V6 region of 16 s rRNA gene representing 789 to 1068 base pairs of *E. coli* genome. The following pair of primers were used: Forward: TAGATACCCSSGTAGTCC (789–806), Reverse: CTGACGRCRGCCATGC (1053–1068). The amplification produces a 279 base pair PCR product. The following amplification condition was used: after an initial denaturation at 95 °C for 10 min, samples were amplified for 35 cycles at 95 °C for 30 s, 55 °C for 30 s and 72 °C for 30 s, and with a final extension at 72 °C for 4 min. The V5-V6 PCR amplicon was gel purified (cat#NA1111, Sigma-Aldrich, USA) and eluted in 40 µl of elution buffer. The concentration of gel eluate was determined by quantifying 1 µl of the eluate by Qubit fluorometer (Invitrogen, Austria) using QuantiFluor ONE dsDNA system (cat#E4871, Promega, USA).

Copy number of PCR amplicons present in nanograms of V5–V6 gel eluate was determined by using the following formula:

$$	\left( {{\mathrm{nanograms}}\,{\mathrm{per}}\,{\mathrm{microliter}}} \right) \times 6.022 \times 1023/\\ 	 \left( {{\mathrm{length}}\,{\mathrm{of}}\,{\mathrm{amplicon}}\,{\mathrm{in}}\,{\mathrm{base}}\,{\mathrm{pairs}}} \right) \times 1 \times 109 \times 650$$

After determining the copy numbers, serial dilutions of the V5–V6 eluate was made to obtain concentrations from 1 × 10^6^ to 1 × 10^1^. These serial diluted samples were then analysed by real time PCR in the presence of QuantiNova SYBR Green PCR Kit (Cat#208052, Qiagen, Germany) in Qiagen 5-plex rotor gene real time PCR system to establish a linear standard graph. The following amplification condition was used: after an initial denaturation at 95 °C for 5 min, the standards were subjected to 40 cycles of amplification at 95 °C for 15 seconds and 60 °C for 30 s. Linear standard curve thus obtained was stored in the system to be used as reference during sample amplification process.

### Statistical analysis

The Normality tests Kolmogorov-Smirnov and Shapiro-Wilks tests results reveal that all variables do not follow Normal distribution. Therefore, non-parametric methods are applied to analyze the data. To compare S1, S2, and Fold difference values between Groups Kruskal–Wallis test is used followed by Bonferroni adjusted Mann–Whitney test for multiple pair wise comparison. Latest version of Statistical Package for the Social Sciences (IBM SPSS Statistics for Windows, Version 23.0, Armonk, NY: IBM Corp. Released 2015) was used to analyze the data and significance level for all tests were fixed as 5% (*α* = 0.05).

## Results

**Fig. 1:**
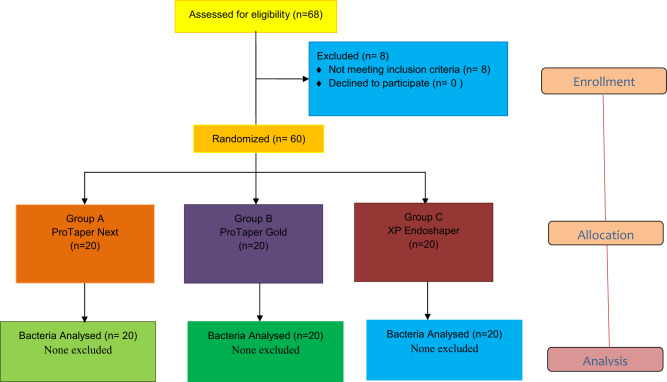
Consolidated standards of reporting trials flowchart. Consolidated standards of reporting trials flowchart showing the flow of participants through each stage of trial.

All sterility control samples were found to be negative for bacteria, therefore none were excluded in this randomized controlled clinical trial. The CONSORT flow chart is given in Fig. 1. All S1 samples from the 60 teeth included in the study were found positive for bacterial presence as revealed by qPCR using universal 16 S rRNA gene-based primers. There was no statistically significant difference between groups in S1 samples (*P* > 0.05). Data from quantitative real time polymerase chain reaction are summarized in Table 1.

**Table 1:** Total bacterial load in root canal samples of teeth with asymptomatic apical periodontitis taken before (S1) and after instrumentation (S2) using ProTaper Next, ProTaper Gold and XP Endoshaper.

Instrumentation groups	Mean (copies/µl)			*p* value	Mean % S1 to S2 reduction
	S1	S2	Fold Difference(S1/S2)		
ProTaper Next	9.94 × 10^7^	11.8 × 10^5^	4.38 × 10^2^	0.012	98.81
ProTaper Gold	20.4 × 10^7^	87.2 × 10^5^	0.80 × 10^2^		95.72
XP Endoshaper	9.20 × 10^7^	4.52 × 10^5^	16.7 × 10^2^		99.50

**Fig. 2:**
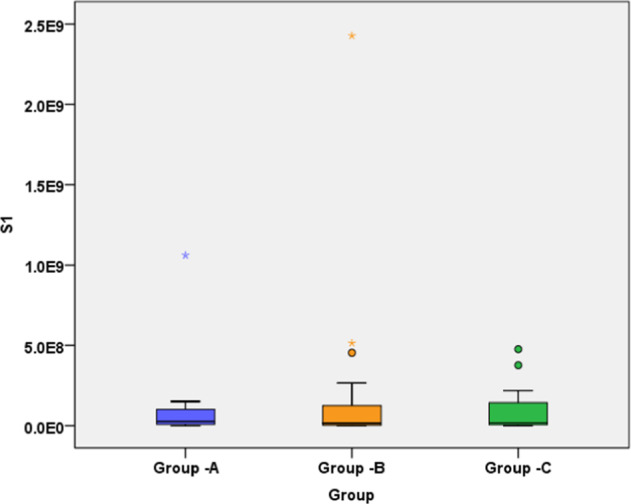
S1 samples. Box plot demonstrating the bacterial load of  samples taken before instrumentation (S1 samples) in each group.

**Fig. 3:**
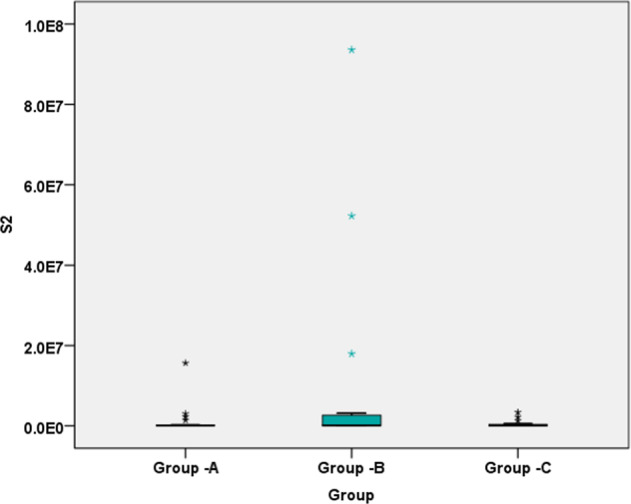
S2 samples. Box plot demonstrating the bacterial load of samples taken after instrumentation (S2 samples) in each group.

**Fig. 4:**
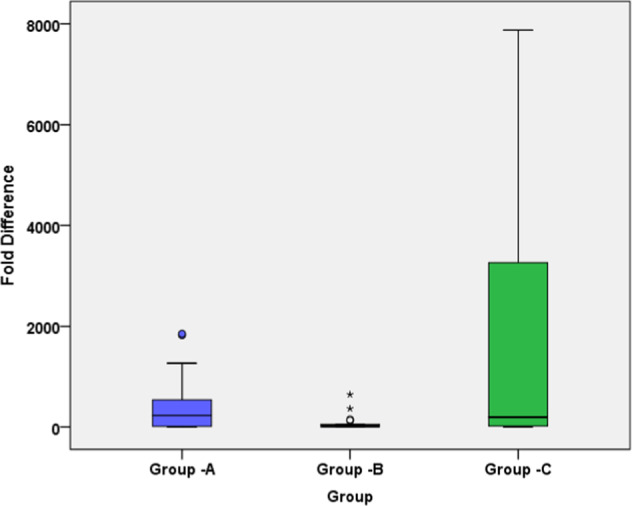
Fold difference. Box plot demonstrating the fold difference (S1/S2) in each group.

In ProTaper Next group, a mean number of 9.94 × 10^7^ bacterial cells occurred in S1 samples. Total bacterial counts were reduced substantially in S2 to a median of 11.8 × 10^5^ cells. The mean fold difference was found to be 4.38 × 10^2^. In ProTaper Gold group, a mean number of 20.4 × 10^7^ cells were present in S1 samples which were reduced to mean number of 87.2 × 10^5^ bacterial cells in S2 samples. The mean fold difference was found to be 0.80 × 10^2^. In XP-endo Shaper group, 9.20 × 10^7^ bacterial cells were found in S1 samples which was significantly reduced to 4.52 × 10^5^ cells in S2 samples. The mean fold difference was found to be 16.7 × 10^2^. S2 quantitative data revealed no statistically significant difference between groups(*P* > 0.05). However, there exist statistically significant differences between ProTaper Gold and XP-endo Shaper (*P* < 0.05) when comparing the fold difference. Box plot values showing median, first quartile & third quartile are summarized in Table 2. Box plot for S1, S2, and Fold difference were depicted in Figs. 2–4.

**Table 2:** Box plot values showing median, first quartile & third quartile.

Instrumentation groups	Median			First quartile			Third quartile		
	S1	S2	Fold difference (S1/S2)	S1	S2	Fold difference (S1/S2)	S1	S2	Fold difference (S1/S2)
ProTaper Next	2.64 × 10^7^	7.03 × 10^4^	2.27 × 10^2^	8.35 × 10^6^	3.30 × 10^4^	1.23 × 10^1^	1.03 × 10^8^	2.24 × 10^5^	5.42 × 10^2^
ProTaper Gold	1.60 × 10^7^	12.0 × 10^4^	0.20 × 10^2^	2.82 × 10^6^	5.31 × 10^4^	0.58 × 10^1^	1.25 × 10^8^	26.47 × 10^5^	0.52 × 10^2^
XP Endoshaper	1.81 × 10^7^	7.54 × 10^4^	1.94 × 10^2^	7.21 × 10^6^	4.60 × 10^4^	1.91 × 10^1^	1.43 × 10^8^	3.33 × 10^5^	32.60 × 10^2^

## Discussion

This double blinded randomized controlled clinical trial followed CONSORT guidelines and compared the effectiveness of ProTaper Next, ProTaper Gold and XP-endo Shaper in eliminating bacteria embedded in root canals of teeth with asymptomatic apical periodontitis. The null hypothesis was rejected as there were significant differences in microbial reduction between groups.

The rotary instruments used in this study were manufactured in different metallurgical phases i.e., ProTaper Next incorporates the M wire technology (Austenitic with small amounts of R-phase and martensite), ProTaper Gold involves a gold heat-treated process and finally XP-endo Shaper encases the MaxWire technology (Martensitic at 20 °C, Austenitic at 35 °C).^28^

The treatment protocol has been standardized in this clinical trial preliminary with a well-trained single operator, straight canals, fixed volume/concentration of irrigant, time of retention and master apical file size ISO diameter 30. Invitro studies have used saline as primary root canal irrigant rather than sodium hypochlorite during root canal preparation to analyse the efficiency of these systems so as to maintain a strategic distance from antimicrobial activity of irrigant.^22,23^ However, the aforesaid studies have suggested use of NaOCl as primary irrigant in future studies to affirm the effect of various systems in clinical scenarios.

Intragroup quantitative analysis revealed that XP-endo Shaper was exceptionally successful in decreasing the intracanal bacterial levels. The mean fold difference of XP Endoshaper was found to be 16.7 × 10^2^ (median, 1.94 × 10^2^), whilst the ProTaper Next demonstrated 4.38 × 10^2^ (median, 2.27 × 10^2^) and ProTaper Gold group showed 0.80 × 10^2^ (median, 0.20 × 10^2^). This was in concurrence with all studies published till date emphasizing that chemo-mechanical debridement is of paramount importance in bringing the infectious bioburden down to controllable levels inside the primary root canals.^29–31^ Henceforth, this clinical trial additionally supports previous studies demonstrating that a large number of cases still harbour noticeable degrees of cultivable microorganisms after instrumentation/irrigation. The rate of cases positive for bacteria in S2 was higher in the more sensitive qPCR analysis. These outcomes confirm the need for supplementary disinfection after chemo-mechanical preparation.

Optimum success of endodontic treatment relies on maximal decrease of bacterial burden to levels compatible with periradicular tissue healing,^32^ therefore it is imperative to utilize highly sensitive methods to quantify reduction in intracanal bacterial populations. For instance, if no system can predictably eliminate all the discernible bacteria from the main root canal (as determined by qualitative analysis), the best one will be the system that advances significantly higher decrease in bacterial levels. Quantitative intergroup correlation between ProTaper Next and ProTaper Gold instrumentation demonstrated no significant difference between them in reducing bacterial burden. There were statistically significant difference between ProTaper Gold and XP Endoshaper.

The percentage total bacterial load reduction of XP-endo Shaper was found to be 99.50% which was higher than the values acquired by ProTaper Next (98.81%) and ProTaper Gold (95.72%). This could be due to 84% of available space inside canal lumina which considers evacuation of enormous amount of debris and avoids its extrusion beyond the apex while being compacted into canal irregularities.^33^ Turbulence generated by the XP Endoshaper could be the conceivable explanation behind the penetration of irrigants in all dentinal tubules.

Results of the present study are in concurrence with previous in vitro studies wherein XP-endo Shaper has shown better intracanal bacterial reduction than WaveOne Gold and Reciproc system using quantitative real time polymerase chain reaction, colony forming units, micro-computed tomographic imaging and scanning electron microscope.^22,23^ The effectiveness of ProTaper Next also has been affirmed by various in vitro studies wherein ProTaper Next advances higher bacterial reduction than Twisted file, PTU and Manual technique In an in vitro study comparing ProTaper Next, PTU and WaveOne in canals contaminated with *E. faecalis*, it was found that ProTaper Next was best in bacterial reduction when compared with PTU and WaveOne.^34^ This demonstrates that instrument design assumes a major role in bacterial reduction during root canal preparation.

The hypothesis that the geometrical design of instrument can impact the decrease of microbial load has been affirmed. The rectangular shaped cross-section of ProTaper Next shapes the root canal asymmetrically, with only two points of contact being available during continuous rotation. Henceforth, dentinal debris can be evacuated coronally when instrument has a larger area of getaway. The dentinal debris compacted onto the root canal walls blocks the dentinal tubules and this might hinder the expulsion of bacteria from inside them. Despite having the greatest number of files, the ProTaper Gold demonstrated the lowest bacterial reduction, perhaps in light of the fact that it has asymmetrical design and a constant pitch.

The mean microbial load reduction percentage from S1 to S2 in our investigation is superior to previous studies where Reciproc, BioRace and hand instrumentation showed 95.9%, 96.9% and 95.2% mean bacterial reduction respectively where they have used 2.5% NaOCl as an irrigant.^35,36^

Our results demonstrated that “not the number, but the design of file system” plays a pivotal role in microbial removal from the root canal system along with the endodontic irrigant.

The major limitation of the present study was the curbed ability of paper points to collect a representative sample from the root canal system, thereby limiting the data on bacterial counts to the main canal only.^37,38^

## Conclusion

XP-endo Shaper was effective in reducing total bacterial load in root canals of teeth with asymptomatic apical periodontitis when compared with ProTaper Gold pointing to the fact that instrument geometry plays a pivotal role in bacterial reduction. ProTaper Next showed better microbial reduction percentage than ProTaper Gold since it maintains a two-point contact with bigger radius on canal walls whereas ProTaper Gold maintains three-point contact with comparatively lesser radius on wall which interferes with removal of bacterial debris from root canal. Therefore, it is important to select the appropriate type of instrument to aid in the chemo-mechanical debridement procedures.

### Future scope

1. Long-term follow-up of these cases is important to consider which system/protocol was most effective.
2. Future studies should consider the efficiency of three-dimensional rotary systems over conventional rotary/reciprocating systems alongside irrigants for choosing the appropriate type of instrument for upgrading disinfection of root canal system in endodontics.
